# Network-based analysis reveals distinct association patterns in a semantic MEDLINE-based drug-disease-gene network

**DOI:** 10.1186/2041-1480-5-33

**Published:** 2014-08-06

**Authors:** Yuji Zhang, Cui Tao, Guoqian Jiang, Asha A Nair, Jian Su, Christopher G Chute, Hongfang Liu

**Affiliations:** 1 Division of Biostatistics and Bioinformatics, University of Maryland Greenebaum Cancer Center and Department of Epidemiology and Public Health, University of Maryland School of Medicine, Baltimore, MD, USA; 2 School of Biomedical Informatics, University of Texas Health Science Center at Houston, Houston, TX, USA; 3 Division of Biomedical Statistics and Informatics, Department of Health Sciences Research, Mayo Clinic, Rochester, MN, USA; 4 Radiology Informatics Laboratory, Department of Radiology, Mayo Clinic, Rochester, MN, USA

## Abstract

**Background:**

A huge amount of associations among different biological entities (e.g., disease, drug, and gene) are scattered in millions of biomedical articles. Systematic analysis of such heterogeneous data can infer novel associations among different biological entities in the context of personalized medicine and translational research. Recently, network-based computational approaches have gained popularity in investigating such heterogeneous data, proposing novel therapeutic targets and deciphering disease mechanisms. However, little effort has been devoted to investigating associations among drugs, diseases, and genes in an integrative manner.

**Results:**

We propose a novel network-based computational framework to identify statistically over-expressed subnetwork patterns, called network motifs, in an integrated disease-drug-gene network extracted from Semantic MEDLINE. The framework consists of two steps. The first step is to construct an association network by extracting pair-wise associations between diseases, drugs and genes in Semantic MEDLINE using a domain pattern driven strategy. A Resource Description Framework (RDF)-linked data approach is used to re-organize the data to increase the flexibility of data integration, the interoperability within domain ontologies, and the efficiency of data storage. Unique associations among drugs, diseases, and genes are extracted for downstream network-based analysis. The second step is to apply a network-based approach to mine the local network structure of this heterogeneous network. Significant network motifs are then identified as the backbone of the network. A simplified network based on those significant motifs is then constructed to facilitate discovery. We implemented our computational framework and identified five network motifs, each of which corresponds to specific biological meanings. Three case studies demonstrate that novel associations are derived from the network topology analysis of reconstructed networks of significant network motifs, further validated by expert knowledge and functional enrichment analyses.

**Conclusions:**

We have developed a novel network-based computational approach to investigate the heterogeneous drug-gene-disease network extracted from Semantic MEDLINE. We demonstrate the power of this approach by prioritizing candidate disease genes, inferring potential disease relationships, and proposing novel drug targets, within the context of the entire knowledge. The results indicate that such approach will facilitate the formulization of novel research hypotheses, which is critical for translational medicine research and personalized medicine.

## Background

A large amount of associations among biomedical entities are scattered in biomedical literature. Systematic analysis of such heterogeneous data provides biomedical scientists with unprecedented opportunities to infer novel associations among different biological entities in the context of personalized medicine and translational research studies. MEDLINE (http://www.nlm.nih.gov/bsd/pmresources.html), for instance, currently contains more than 22 million citations of biomedical literature. Semantic MEDLINE is a knowledge base consisting of associations automatically extracted from MEDLINE by integrating document retrieval, advanced natural language processing (NLP), and automatic summarization and visualization [1]. However, it is computationally challenging to perform queries directly from Semantic MEDLINE where associations among different biomedical entities are very complex yet sparse. It is also very difficult to investigate those associations at a large scale. Advance informatics approaches have the potential to fill gaps between knowledge needs of translational researchers and existing knowledge discovery services.

In Semantic MEDLINE, biomedical entities and associations are semantically annotated using concepts in the Unified Medical Language System (UMLS) [2]. The semantic information defined in the UMLS can be further leveraged to extract associations among concepts in specific domains and identify domain patterns for specific studies through advanced computational methods such as network-based analysis.

In the last decade, network-based computational approaches have gained popularity and become a new paradigm to investigate associations among drugs, diseases, and genes. Applications of these approaches include disease gene prioritization [3-5], identification of disease relationships [6,7] and drug repositioning [8,9]. However, majority of these approaches focus on relationships between only two kinds of entities (e.g., association between gene and disease). For instance, Hu and Agarwar [10] created a human disease-drug network based on genomic expression profiles collected from the Gene Expression Omnibus (GEO) (http://www.ncbi.nlm.nih.gov/geo/). In total, 170,027 interactions between diseases and drugs were considered significant, including 645 disease-disease, 5,008 disease-drug, and 164,374 drug-drug associations. These expression-based associations among diseases and drugs could serve as a backend knowledge base to facilitate discovery. Bauer-Mehren et al. [11] developed a comprehensive disease-gene association network by integrating associations from several sources that cover different biomedical aspects of diseases. The results indicate a highly shared genetic origin of human diseases. Functional modules were also detected in several Mendelian disorders as well as in common diseases. To systematically analyze drug-disease-gene relationships, Daminelli et al. [12] proposed a network-based approach to predict novel drug-gene and drug-disease associations by completing incomplete bi-cliques in the network. This approach holds great potential for drug repositioning and discovery of novel associations. However, the analysis was limited to only certain associations among drugs, genes, and diseases (e.g., drug-disease and drug-gene associations). A network-based investigation of all pair-wise associations among these entities is necessary to understand the complexity of existing associations and to infer novel associations within the context of the whole knowledgebase.

Network-based computational approaches enable us to analyze heterogeneous networks such as drug-disease-gene networks by decomposing them into small subnetworks, called network motifs (NMs) [13]. NMs are statistically significant recurring structural patterns found more often in real networks than would be expected in random networks with the same network topologies. They are the smallest basic functional and evolutionarily conserved units in biological networks. Our hypothesis is that NMs of a network are the significant sub-patterns that represent the backbone of the network, which serves as the focused portion out of thousands of nodes (e.g., drugs, diseases, and genes,) [14,15]. These NMs could also form large aggregated modules that perform specific functions by forming associations in overlapping NMs.

In this paper, we propose a network-based computational framework to analyze the complex network formed by a large amount of associations. We focus on a heterogeneous drug-disease-gene network derived from Semantic MEDLINE and investigated underlying associations using network-based systems biology approaches. Three case studies demonstrate that our approach has potential to facilitate formulization of novel research hypotheses, which is critical for translational medicine research. In the following, we first present Materials and methods. We then describe the results and case studies in detail.

## Materials and methods

**Figure 1:**
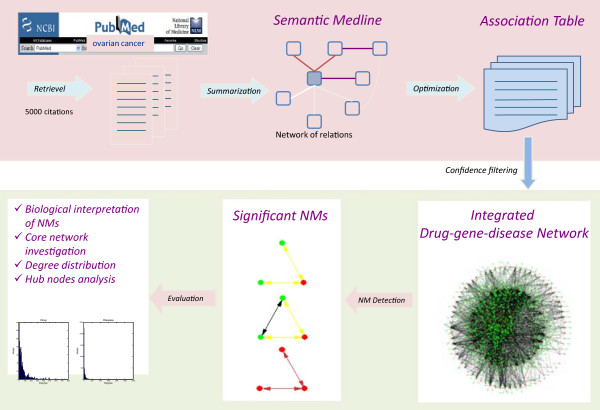
Overview of the network-based computational framework for an integrated drug-disease-gene network.

To comprehensively investigate the integrated drug-disease-gene network formed by associations available in Semantic MEDLINE, we propose the following two-step computational framework: (1) extraction and optimization of drug-disease-gene network in Semantic MEDLINE; (2) network topology analysis of this heterogeneous network at two levels: statistics and degree distribution of high-confidence association networks, and distinct pattern detection at NM level. In this section, we first describe the steps to extract association network data from MEDLINE database, followed by a description of the proposed network-based approach to investigate this heterogeneous drug-disease-gene association network. Figure 1 illustrates the steps of the proposed approach.

### Data sources and preprocessing

#### Extraction of association data from Semantic MEDLINE

Semantic MEDLINE currently contains more than 56 million associations extracted from MEDLINE citations and consists of eight tables, including concepts, concept semantic types, concept translations, predication, predication arguments, and sentences. Data from different tables need to be joined in order to obtain information for a particular association between two entities. The database contains an all-embracing joined table that provides information about associations (source concept, predicate, and object concept), and their source PubMed IDs (PMIDs).

We optimize and reorganize the relevant data in Semantic MEDLINE into the Resource Description Framework (RDF) format. Based on the UMLS semantic types and groups [16], we extract unique associations among drugs, diseases, and genes, and represent them in six views in relational database tables. We then use the Web RDF transformation tool D2R server to convert the six views into RDF triples through a D2RQ mapping file (http://d2rq.org/d2r-server). This mapping file specifies the mappings between those six relational database table schemas and the output RDF graphs [17]. A detailed description of this approach is described in our previous work [18]. These six tables are used as preliminary association data resources including all unique associations from Semantic MEDLINE.

#### Data preprocessing using FDA-approved drugs in DrugBank

Since the extraction accuracy of associations in Semantic MEDLINE is about 77% (precision is 76% to 96%, and recall is 55-70%) [19], a filtering strategy is applied to extract high-confidence association data using the FDA-approved drug list from DrugBank, a database containing drug information and the corresponding drug target and treatment indication information [20]. As of July 31 2012, the database contains 1,578 FDA-approved drug entries, including 131 FDA-approved biotech drugs, and 1,447 FDA-approved small molecule drugs. We extract associations involving these FDA-approved drugs from each drug-related association table. After manually removing generic and nonsensical terms in the association tables (e.g., gene, homologous gene, and protein), we limit the drug-drug, drug-gene, and drug-disease associations to those involved in the 1,578 FDA-approved drugs. Based on the filtered drug-gene and drug-disease associations, we generate related gene and disease lists and then obtained gene-gene, disease-disease, and gene-disease associations using these genes and diseases. This filtering strategy enables us to focus on associations related to FDA-approved drugs only in this study. These associations are then analyzed by the proposed network-based approach.

### Network motif analysis

Network motifs are topologically distinct subnetwork patterns that are present more frequently in true networks than in random networks [21]. They are usually well conserved and possess specific processing tasks in same types of networks. For example, in gene regulatory networks, the same set of network motifs have been repeatly identified in diverse organisms from bacteria to human [22]. The hypothesis is that network motifs were independently selected by evolutionary processes in a converging manner and have characteristic dynamical functions [23]. This suggests that network motifs serve as building blocks of in gene regulatory networks that are beneficial to the organism.

In this study, we extend network motif analysis to the disease-drug-gene network. Six different types of associations among drugs, diseases, and genes are integrated into a heterogeneous disease-drug-gene network. In this network, nodes represent biomedical entities stored in the RDF triples (i.e., diseases, drugs, or genes in “subject” and “object”), and edges represent associations between two biomedical entities (i.e., relationships in “predicate”). For simplicity, we consider all associations as undirectional association relationships in this study, discarding the directionality and types in the original RDF graph. In other words, as long as there is an association between two nodes, we consider there is an edge between these two nodes. We hypothesize that even within such simplified disease-drug-gene association network, network motifs in the network can (1) represent basic inter-relationships among diseases, drugs, and genes; (2) reflect a framework in which particular functions are achieved efficiently. Specifically, we focus on three-node network motifs in this disease-drug-gene network since they are the building blocks for larger size network motifs (number of nodes > 3) [24]. All connected subnetworks containing three nodes in the association network are collated into isomorphic patterns [25], and the frequency of the patterns are counted. We also generated 1000 random networks from the original network by switching edges between vertices and preserving the number of edges between types of nodes (i.e., disease, drug and gene). By the default of FANOMD algorithm, if the number of occurrences for each pattern is at least five in the real network, which is significantly higher than randomized networks, the pattern is considered to be a network motif. Statistical significance test is performed by computing the fraction of randomized networks in which the pattern appears at least as often as in the interaction network [24]. The *z* score is calculated using the following equation:

(1) $$Z=\frac{N_{\mathrm{real}}-\langle N_{\mathrm{rand}}\rangle }{\sigma_{\mathrm{rand}}}$$

where *N*_*real*_ is the number of times one three-node subnetwork is detected in the real network, *N*_*rand*_ is the mean number of times this subnetwork is detected in 1000 randomized networks, and σ_*rand*_ is the standard deviation of the number of times this subnetwork is detected in randomized networks. The *p* value of a motif is the number of random networks in which it occurs more often than in the original networks, divided by the total number of random networks. A pattern with *p* ≤ 0.05 is considered statistically significant. This network motif discovery procedure is performed using the FANMOD tool [26].

### Construction of the core drug-disease-gene network

It has been shown that in gene regulatory networks, for each network motif, the majority of matches overlap and aggregate into homologous motif clusters [27]. Many of these motif clusters largely overlap with modules of known biological processes [28]. The clusters of overlapping matches of these motifs aggregate into a superstructure that presents the backbone of the network and is assumed to play a central role in defining the global topological organization. Accordingly, we aggregate matches of significant network motifs into a core drug-disease-gene network. In this core network, we investigate the distribution of the connectivity degree of different types of nodes. Nodes with significantly larger number of links in the network are called hub nodes, which is critical in the information flow exchange throughout the entire network.

## Results

### An integrated drug-disease-gene network reconstructed from Semantic MEDLINE

We constructed a drug-disease-gene network with the following two steps:

First, we extracted unique association data from Semantic MEDLINE. Using a use-case driven database optimization approach developed in our previous work [18], we extracted six different types of associations from Semantic MEDLINE database. Table 1 shows basic statistics of these six groups of associations. As illustrated in Table 1, the number of unique associations (the Unique Association column) for each type of associations is significantly less than the number of total associations (the Record column). Since the prediction accuracy of Semantic MEDLINE is approximately 77% [29], we used a filtering strategy to focus on associations involving FDA-approved drugs for downstream network-based analysis.

**Table 1:** Statistics of the six extracted association types

**Association type**	**Record in Semantic MEDLINE**	**Unique associations**	**Associations involving FDA-approved drugs**	**Unique entity number**
Disease-Disease	2,516,049	843,221	1684	2,248
Disease-Gene	206,155	111,117	21,444	5,954
Disease-Drug	3,021,256	1,277,879	54,996	3,414
Drug-Gene	398,572	248,491	3758	1,451
Drug-Drug	4,780,394	1,900,576	266	382
Gene-Gene	108,035	49,593	2169	2,792
Total	11,030,461	4,430,877	84,317	7,243^1^

^1^This is the unique number of entities by summarizing all the associations.

Second, we constructed association related data involving FDA-approved drugs. We applied the filtering strategy discribed in the Materials and methods section to extract association data involving FDA-approved drugs from the unique association data set. As shown in the “Associations Involving FDA-approved Drugs” column in Table 1, the association number of each table was further reduced. We used this focused association data to construct an integrated disease-drug-gene network for downstream network-based analysis.

### Network topology analysis of the core drug-disease-gene network

**Figure 2:**
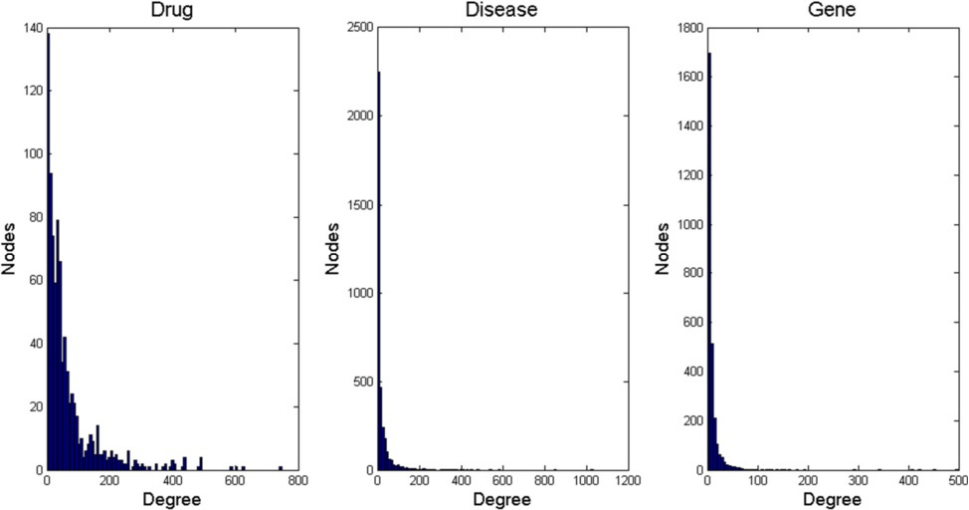
Degree distribution of three biomedical entities: drug, gene, and disease.

The network motif analysis was performed on the integrated disease-drug-gene network obtained in Section An integrated drug-disease-gene network reconstructed from Semantic MEDLINE. Since the network contains thousands of associations among 865 drugs, 2791 genes, and 3578 diseases (Table 1), it is too complex for a direct visualization. We overcame this problem by identifying enriched network motifs and interpreting them through an enhanced visualization. Out of this heterogeneous network consisting of 84,317 associations among 7,234 entities (including drugs, diseases, and genes), five significant network motifs were identified. Figure 2 presents detailed statistics on these network motifs. The matches of these network motifs were extracted and number of matches for each network motif was counted (“Number of Matches” column in Figure 2).

**Figure 3:**
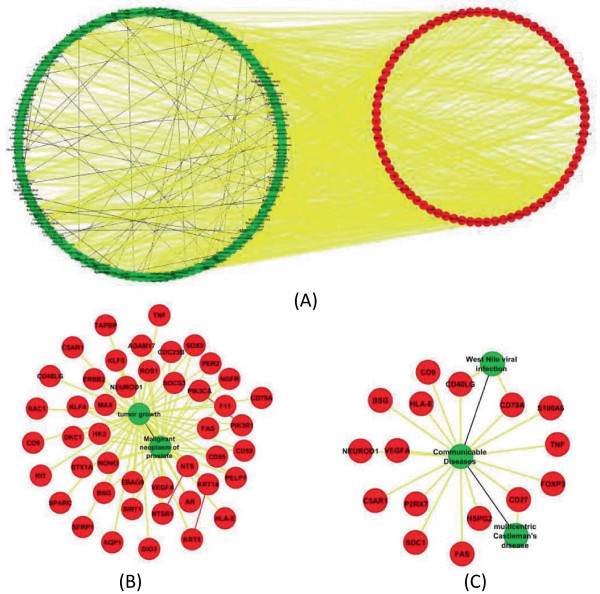
**Subnetworks extracted from NM 1. (A)** Overview of the subnetwork, consisting of 126 diseases and 79 genes. **(B)** Subnetwork associated with “Malignant neoplasm of prostate” and “tumor growth”. **(C)** Subnetwork associated with “communicable diseases”, “West Nile viral infection” and “multicentric Castleman's disease”.

Based on the network motifs identified in the analysis, we constructed a core disease-drug-gene network aggregated from significant network motif instances. We then investigated the degree distribution of different types of entities in the integrated network. Figure 3 represents the degree distribution of disease, drug, and gene nodes in the core drug-disease-gene network. All three distributions follow the power-law distribution, indicating that networks related to different types of nodes are scale-free. The majority of the nodes in the network have only a few (less than 10) links but few other nodes have a large number of links. Such distributions have been observed in many studies of biological networks [30]. Our analysis demonstrates for the first time that in an integrated network consisting of heterogeneous associations, the scale-free network structure still holds. The hub nodes (i..e, the nodes have a large number of links) can provide scientists future research directions.

### Local network structure: from network to network motif

The five significant network motif patterns in Figure 2 have strong biological meanings and could suggest scientists future directions in their research field. We provided three case studies in the following sections to illustrate results based on three significant network motifs.

#### Case study 1 - prioritization of disease genes

**Figure 4:**
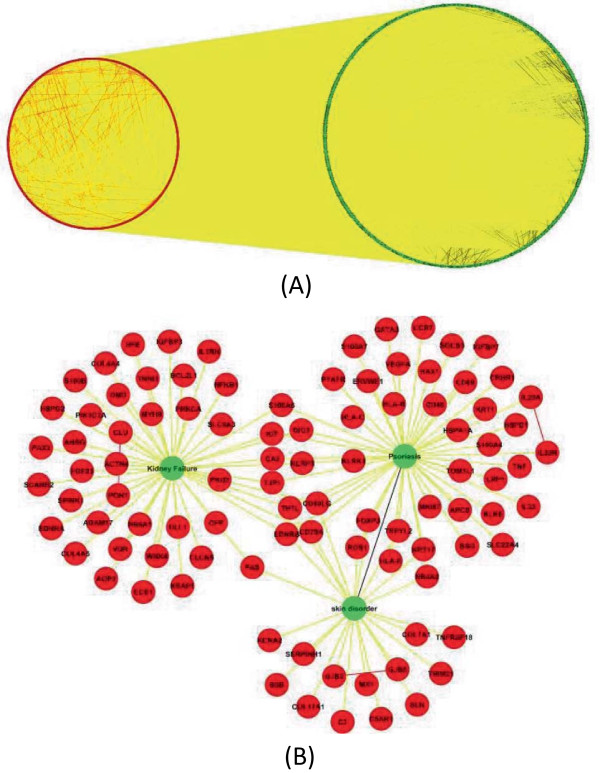
**Subnetworks extracted from NM 4. (A)** Overview of the subnetwork, consisting of 2,664 diseases and 1,122 genes. **(B)** Subnetwork associated with “Kidney Failure” and “skin disorder”.

We first investigated whether the network motif analysis could help prioritize disease genes based on the associations between diseases and their surrounding genes. One example is Network Motif 1 (NM 1) in Figure 2, in which two diseases that are associated with each other are also associated with one common disease gene. This indicates that diseases identified to be associated in literature are more likely to share same associated disease genes. To further investigate the relationships highlighted by NM 1, We extracted all associations relationships among 126 diseases and 79 genes in NM 1. In total, there are 71 disease-disease, 853 disease-gene, and 3 gene-gene associations (Figure 4(A)) in this subnetwork, suggesting that diseases that are associated with each other are more likely to associate with a group of common disease genes. For instance in Figure 4(B), “Malignant neoplasm of prostate” shares all 35 associated genes with “tumor growth”. Similar findings have also been discovered in other studies demonstrating same functional modules/pathways being affected in similar diseases [6,31,32]. There are 10 genes only associated to “tumor growth” in literature. Such information will help scientists generate testable hypotheses of possible roles of these genes in prostate cancer research. Another example is shown in Figure 4(C), where “communicable diseases” was identified to have common associated genes with both “West Nile viral infection” and “multicentric Castleman’s disease”. Thirteen genes associated only with “communicable diseases” can be considered as candidate disease genes for the other two diseases and help scientists design future exploratory experiments. The detailed network information is presented in Additional file 1: File S1.

#### Case study 2 - inference of disease relationships

**Figure 5:**
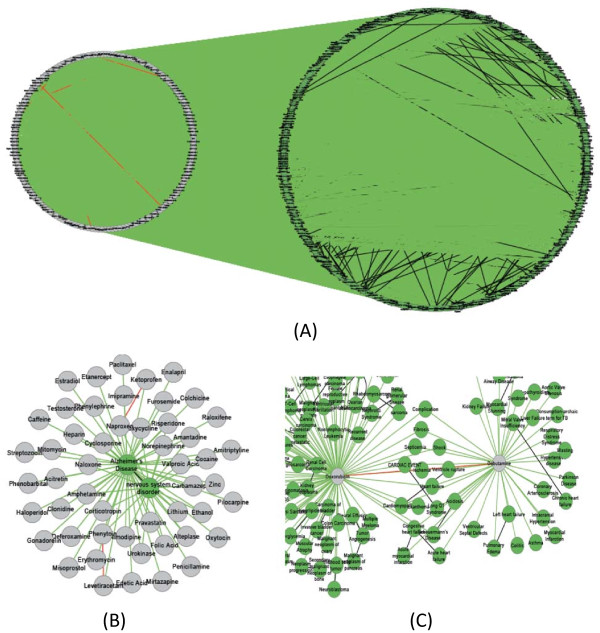
**Subnetworks extracted from NM 2. (A)** Overview of the subnetwork, consisting of 468 disease and 162 drugs.** (B)** Subnetwork associated with “Alzheimer’s Disease” and “nervous systems disorder”. **(C)** Subnetwork associated with “Dobutamine” and “Doxorubicin”.

Very interestingly, we also identified another similar disease-gene network motif in our analysis (NM 4). The only difference between NM 1 and NM 4 is that NM 4 doesn’t have the associations between two diseases themselves. We extracted all associations among 2,664 diseases and 1,122 genes in NM 4. In total, there are 860 disease-disease, 17,242 disease-gene, and 310 gene-gene associations in this subnetwork (Figure 5(A)). Based on the “guilt by association” rule – diseases similar to each other are more likely to be affected by the same genes/pathways, two diseases involved in the same NM 4 are more likely to be similar/associated than other diseases [6]. For instance in Figure 5(B), “Kidney Failure” and “skin disorder” are associated with a group of five common associated genes. A wide variety of different skin disorders have been observed in patients with kidney diseases [33]. One example is the “psoriasis” disease. During the treatment of psoriasis with fumaric acid derivatives, patients could develop acute kidney failure [34]. In the subnetwork that consists of first neighbors of these two diseases, psoriasis is also included and has common associated genes with both “kidney failure” and “skin disorder”. Some genes in the network are associated with one of these diseases only but not both. To investigate enriched biological functions/processes, we performed functional enrichment analysis on neighbor genes of three diseases with Ingenuity Pathway Analysis (IPA) Suite (http://www.ingenuity.com/). These genes are enriched in kidney-related disease categories (Table 2). Although a major portion of neighbor genes are related to “skin disorder” or “psoriasis” only, they have been annotated with kidney related dysfunctions in the IPA database. Given the fact that associations among thousands of diseases are complex yet incomplete, the inferred association relationships based on our network motif-based analysis can mine the significant network topology properties of association networks and guide scientists to investigate significant association relationships in future experiments. The detailed network information is presented in Additional file 2: File S2.

**Table 2:** Enriched disease and disorder categories in IPA analysis

**Category**	**p-value**	**Molecules**
Renal Inflammation	6.62E-09	VEGFA,COL4A5,CD40LG,APCS,IL1RN,CLU,MYH9,COL4A4,VDR,ACTN4,NFKB1,TNF,FAS
Renal Nephritis	6.62E-09	VEGFA,COL4A5,CD40LG,APCS,IL1RN,CLU,MYH9,COL4A4,VDR,ACTN4,NFKB1,TNF,FAS
Congenital Heart Anomaly	3.41E-06	VEGFA,HSPG2,TRIM21,EDNRA,ECE1
Liver Cirrhosis	4.13E-06	ADAM17,CD40LG,C5AR1,EDNRB,BSG,PTAFR,TNF,CCR7
Glomerular Injury	5.22E-06	VEGFA,CLU,MYH9,ACTN4
Cardiac Infarction	6.38E-06	PON1,BCL2L1,CD40LG,IL1RN,HSPA1A/HSPA1B,CLU,TNNI3,TNF,LRP1
Renal Atrophy	7.66E-06	CD40LG,EDNRB,FGF23,EDNRA,VDR,AQP2
Liver Damage	9.94E-06	BCL2L1,NLRP3,BSG,IL1RN,NFKB1,TNF,FAS
Liver Proliferation	1.75E-05	VEGFA,SOCS3,EDNRB,IL1RN,EDNRA,NFKB1,TNF,FAS
Pulmonary Hypertension	3.13E-05	EDNRB,IL1RN,KIT,EDNRA
Liver Hepatitis	4.73E-05	BCL2L1,IL23A,TNF,CCR7,FAS
Liver Necrosis/Cell Death	6.57E-05	SOCS3,BCL2L1,CD40LG,IL1RN,HSPD1,NFKB1,TNF,FAS
Cardiac Inflammation	6.64E-05	IL33,CLU,TNNI3,IL23A,NFKB1,TNF
Heart Failure	6.76E-05	BCL2L1,CA2,TNNI3,VDR,NFKB1,TNF,AQP2,PRKCA
Hepatocellular Carcinoma	6.87E-05	VEGFA,CA2,BCL2L1,SOCS3,ADAM17,BSG,KEAP1,CLU,IGFBP3,S100A4,KIT,MKI67,TNF
Liver Hyperplasia/Hyperproliferation	6.87E-05	VEGFA,CA2,BCL2L1,SOCS3,ADAM17,BSG,KEAP1,CLU,IGFBP3,S100A4,KIT,MKI67,TNF
Renal Dysfunction	2.46E-04	BSG,FGF23,TNF
Cardiac Necrosis/Cell Death	3.17E-04	VEGFA,SOCS3,BCL2L1,S100B,HSPD1,TNF,LRP1,NAD+
Cardiac Hypertrophy	5.67E-04	IL33,ADAM17,S100A6,HSPA1A/HSPA1B,FGF23,EDNRA,DMD,VDR,NFKB1,TNF,PRKCA
Renal Necrosis/Cell Death	5.83E-04	BCL2L1,HSPA1A/HSPA1B,IGFBP3,CLU,PAX2,NFKB1,TNF,FAS,PRKCA
Liver Inflammation	8.62E-04	IL1RN,FOXP3,NFKB1,TNF,FAS
Kidney Failure	1.37E-03	VEGFA,SLC9A3,PKD2,MYH9,VDR,TNF,AQP2
Cardiac Proliferation	1.66E-03	ADAM17,KIT,TNF,PRKCA
Renal Dilation	1.67E-03	EDNRB,EDNRA,AQP2
Nephrosis	2.35E-03	CLU,ACTN4
Liver Fibrosis	2.48E-03	VEGFA,SOCS3,EDNRB,PKD2,EDNRA,NFKB1,TNF,CCR7
Renal Proliferation	2.48E-03	SOCS3,HSPG2,TJP1,HSPD1,TNF,CCR7
Increased Levels of AST	3.13E-03	TNF,FAS
Cardiac Fibrosis	4.69E-03	TNNI3,DMD,VDR,NFKB1,TNF,DIO3
Increased Levels of Albumin	5.63E-03	VEGFA
Liver Regeneration	5.85E-03	SOCS3,IL1RN,TNF

#### Case study 3 – Drug repositioning

**Figure 6:**
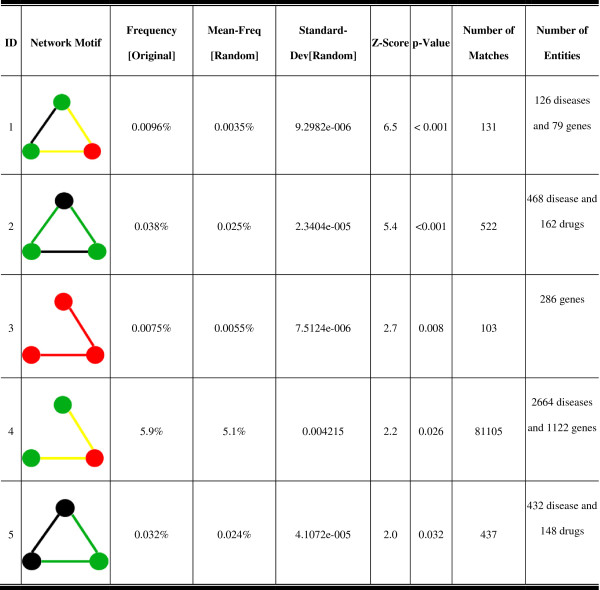
**Statistics of significant network motifs.** Node color: black – drug, green – disease, red – gene. Edge color denotes the associations between different biomedical entities: black – association between disease and disease, yellow - association between disease and gene, green - association between disease and drug, red - association between gene and gene.

Network Motif 2 (NM 2) suggests another association pattern between diseases and drugs, in which two diseases associated with each other are targets for the same drug. It has been shown by Suthram et al. [7] that diseases with significant correlations based on mRNA gene expression data also share common drugs. This NM supports the hypothesis that similar diseases can be treated by same drugs, allowing us to make hypotheses for drugs repositioning purpose. We extracted all associations among 468 disease and 162 drugs in NM 2. In total, there are 279 disease-disease, 8,730 disease-drug, and 14 drug-drug associations in this subnetwork (Figure 6(A)). We further investigated whether any drugs or diseases were “hub” nodes in this subnetwork. In Figure 6(B), “Alzheimer’s Disease” and “nervous systems disorder” are hub diseases surrounded by 51 FDA-approved drugs. Both diseases are associated with 20 common drugs, while “nervous systems disorder” has associations with additional 31 drugs. These drugs can be considered repositioned for treatment of “Alzheimer’s Disease” since it is a central nervous system disorder characterized by the presence of neurofibrillary tangles, neuritic plaques and dystrophic neurites in the brain [35]. In Figure 6(C), we observed two “hub” drugs surrounding by 129 diseases, 16 of which have associations with both drugs. Dobutamine is a sympathomimetic drug used in the treatment of heart failure and cardiogenic shock. Doxorubicin is a drug used in cancer chemotherapy. Chemotherapy side effects may increase the risk of heart disease in cancer patients [36]. This series of underlying connections can provide clinicians potential side effects related to certain drug treatment. This could take years to study in the clinic to identify such side effects. The results derived from our approach can serve as *in silico* exploratory analysis to guide such studies. The detialed network information is presented in Additional file 3: File S3.

Three-gene network motif (NM 3) was also identified in this heterogeneous network. This NM is a very common motif pattern in the protein-protein interaction network or gene regulatory network [37,38], indicating that NM detection analysis of heterogeneous networks can identify significant NMs even enriched in a single type of associations in a heterogeneous association network.

### Comparisons of network motifs from different networks

Since all five network motifs identified involve only two out of three node types, we further investigated whether the networks involving only two node types can generate the same NMs. To accomplish that, we performed NM analysis on disease-gene, disease-drug and gene networks respectively. Not all NMs detected in the complete network can be detected in disease-gene, disease-drug and gene networks respectively (Additional file 4: File S4). The results indicate that although the NMs don’t contain all three different node types due to small NM size, the additional associations still introduce additional information in the NM detection analysis.

## Discussion

Literature mining approaches have been successful to extract associations among biological entities in the last decade. However, such information is usually large, complex and multidimentional, making it impossible for biomedical researchers to directly investigate such data. To leverage the gap between knowledge needs of translational researchers and existing knowledge discovery services, we have proposed a network-based informatics approach to investigate the underlying relationships among different biological entities based on associations automatically extracted from literature. The proposed approach has advantages in several aspects.

Our approach is one of the first attempts to investigate the disease-drug-gene associations in an integrative manner. To demonstrate the superiority of NM analysis on the heterogeneous network, we performed NM analysis on disease-gene, disease-drug and gene networks respectively and compared results with the ones derived from the complete disease-drug-gene network. Not all network motifs detected in the complete network can be detected in disease-gene, disease-drug and gene networks respectively. The results indicates that although NMs doesn’t contain all three different node types due to their small size in this study, the additional associations still introduce additional information in the analysis. In addition, NM analysis of such heterogeneous networks can extract and highlight the hotspots in the network, leading experts in different fields to generate testable hypotheses in their future research.We are aware that there are many other network analysis approaches for both social networks and biological networks. These approaches are designed for different purposes. For instance, biological networks can be interrogated by their overall properties (e.g., average clustering coefficient and overall distributions of node degrees), significant NMs, or clustered subnetworks/modules. In this work, we focus on identifying statistically significant three-node NM patterns that can help infer novel disease-drug-gene relationships. The NM analysis can decompose the whole heterogeneous network into smallest network patterns that recurrently discovered in the network, considered as the backbone associations of diseases, drugs, and genes. For instance, in NM 1 instances in Figure 2, most of these NMs contain the first two same diseases, while the third gene is different. By extracting all the associations involving these two diseases from the original association network, we found that while these two diseases share a significant number of associated genes, they also have some unique associations with other genes respectively. Based on the assumption that similar diseases are more likely to associate with same group (s) of genes or involve same biological processes, the genes associated only with one disease can be prioritized as candidate disease genes of the second disease. Such inference could only be possible through NM level analysis by considering significant network patterns (i.e., NMs) as well as their neighborhood in the whole network. In addition, since these NMs are statistically significant subnetworks, they represent the “real” signal from the network which usually contains considerable amount of false positive associations, especially those from literature mining techniques. Due to the limitation of computational resource, we didn’t include the NMs with more than three nodes. We plan to extend our work to NMs with more nodes (i.e., >3) when the computational resource become available. We believe that the proposed network-based approach can complement other existing network analysis methods and provide researchers a unique way to look at these huge heterogeneous networks.

From our preliminary study [18], we found that Semantic MEDLINE lacks of gene-gene associations since such information usually are illustrated in the main text of literature. Semantic MEDLINE contains gene-gene interaction data from PubMed literature abstracts (Figure 2). We included all the associations in Figure 2 in our analysis. However, the number of gene-gene association in Semantic MEDLINE (2,169 high-confidence pairs) is relevantly small comparing to other public databases (e.g., HPRD [4]). For instance, we compared the gene-gene associations in Semantic MEDLINE with those in HPRD, a manually curated gene-gene association database in human [4]. The overlap between these two databases is very small (about 10% associations of Semantic MEDLINE can be found in HPRD). HPRD contains many more associations than Semantic MEDLINE (41,327 versus 2,169). Therefore, we believe that combining Semantic MEDLINE with other public resources (such as HPRD [39] and STRING [40]) will increase the coverage of associations and build a more comprehensive association database. Using linked data approach, it will be relatively easier to link our data graph with such databases.

## Conclusions and future work

In this paper, we proposed a network-based computational framework to investigate integrated heterogeneous network extracted from MEDLINE literature, including associations among three major entity categories: drug, gene, and disease. Five significant NMs were identified and considered as the backbone of the entire network. The potential biological meanings of each network motif were further investigated. The results demonstrated that the proposed approach holds the potential to 1) prioritize candidate disease genes, 2) identify potential disease relationships, and 3) propose novel drug targets, within the context of the entire knowledge. We believe that such analyses can facilitate the process of inferring novel relationships between drugs, genes, and diseases. One future direction is to develop module-based approaches to understand associations between different biomedical entities. Modules are condensed subnetworks in a network. Modules identified in heterogeneous networks are a group of related diseases, drugs and genes, which gives researchers a focused network view of the association relationships among these entities. Topology analysis of heterogeneous networks using graphic theory can also be applied in future studies, which can lead to the identification of diseases/drugs/genes in the context of association networks. Pathway level information could also be integrated in future analyses to extend current association network.

## Competing interests

The authors declare that they have no competing interests.

## Authors’ contributions

YZ and CT led the study design and analysis, and drafted the manuscript. GJ contributed to the manuscript preparation and use case discussions. JS and AAN contributed to the association data extraction and reorganization, CGC and HL participated the design, provided support and manuscript editing. All authors read and approved the final manuscript.

## Supplementary Material

Additional file 1 **Detailed network information derived from NM 1 (Figure **4**).** Click here for file

Additional file 2 **Detailed network information derived from NM 4 (Figure **5**).** Click here for file

Additional file 3 **Detailed network information derived from NM 2 (Figure **6**).** Click here for file

Additional file 4 NM analysis of drug-gene, disease-drug, and gene-drug networks. Click here for file

## References

[B1] Rindflesch TC Kilicoglu H Fiszman M Rosemblat G Shin D Kilicoglu H Fiszman M Rosemblat G Shin D Semantic MEDLINE: an advanced information management application for biomedicine Information Services & Use 2011 31 1/2 15 21

[B2] Unified Medical Language System® (UMLS) Available from: http://www.nlm.nih.gov/research/umls/

[B3] Piro RM Di Cunto F Computational approaches to disease-gene prediction: rationale, classification and successes FEBS J 2012 279 5 678 696 10.1111/j.1742-4658.2012.08471.x 22221742

[B4] Kohler S Bauer S Horn D Robinson PN Bauer S Horn D Robinson PN Walking the interactome for prioritization of candidate disease genes Am J Hum Genet 2008 82 4 949 958 10.1016/j.ajhg.2008.02.013 18371930 PMC2427257

[B5] Chen J Aronow BJ Jegga AG Disease candidate gene identification and prioritization using protein interaction networks BMC Bioinformatics 2009 10 73 10.1186/1471-2105-10-73 19245720 PMC2657789

[B6] Goh KI Cusick ME Valle D Childs B Vidal M Barabasi AL The human disease network Proc Natl Acad Sci U S A 2007 104 21 8685 8690 10.1073/pnas.0701361104 17502601 PMC1885563

[B7] Suthram S Dudley JT Chiang AP Chen R Hastie TJ Butte AJ Dudley JT Chiang AP Chen R Hastie TJ Butte AJ Network-based elucidation of human disease similarities reveals common functional modules enriched for pluripotent drug targets PLoS Comput Biol 2010 6 2 e1000662 10.1371/journal.pcbi.1000662 20140234 PMC2816673

[B8] Arrell DK Terzic A Network systems biology for drug discovery Clin Pharmacol Ther 2010 88 1 120 125 10.1038/clpt.2010.91 20520604

[B9] Dudley JT Deshpande T Butte AJ Exploiting drug-disease relationships for computational drug repositioning Brief Bioinform 2011 12 4 303 311 10.1093/bib/bbr013 21690101 PMC3137933

[B10] Hu G Agarwal P Human disease-drug network based on genomic expression profiles PLoS One 2009 4 8 e6536 10.1371/journal.pone.0006536 19657382 PMC2715883

[B11] Bauer-Mehren A Bundschus M Rautschka M Mayer MA Sanz F Furlong LI Bundschus M Rautschka M Mayer MA Sanz F Furlong LI Gene-disease network analysis reveals functional modules in mendelian, complex and environmental diseases PLoS One 2011 6 6 e20284 10.1371/journal.pone.0020284 21695124 PMC3114846

[B12] Daminelli S Haupt VJ Reimann M Haupt VJ Reimann M Schroeder M Schroeder M Drug repositioning through incomplete bi-cliques in an integrated drug-target-disease network Integr Biol (Camb) 2012 4 7 778 788 10.1039/c2ib00154c 22538435

[B13] Milo R Itzkovitz S Kashtan N Levitt R Shen-Orr S Ayzenshtat I Sheffer M Alon U Itzkovitz S Kashtan N Levitt R Shen-Orr S Ayzenshtat I Sheffer M Alon U Superfamilies of evolved and designed networks Science 2004 303 5663 1538 1542 10.1126/science.1089167 15001784

[B14] Zhang Y Xuan J Xuan J Reyes BGDl Reyes BGDl Clarke R Ressom HW Clarke R Ressom HW Reconstruction of gene regulatory modules in cancer cell cycle by multi-source data integration PLoS One 2010 5 4 e10268 10.1371/journal.pone.0010268 20422009 PMC2858157

[B15] Zhang Y Xuan J Xuan J Reyes BG d l Reyes BG d l Clarke R Ressom HW Clarke R Ressom HW Reverse engineering module networks by PSO-RNN hybrid modeling BMC Genomics 2009 Suppl 1 S15 19594874 10.1186/1471-2164-10-S1-S15 PMC2709258

[B16] The UMLS Semantic Groups 2012 Available from: http://semanticnetwork.nlm.nih.gov/SemGroups/

[B17] RDF graph definition cited 2012; Available from: http://www.w3.org/TR/rdf-mt/#graphdefs

[B18] Tao C Zhang Y Jiang G Bouamrane M-M Chute CG Zhang Y Jiang G Bouamrane M-M Chute CG Optimizing semantic MEDLINE for translational science studies using semantic web technologies, in Proceedings of the 2nd international workshop on Managing interoperability and compleXity in health systems 2012 Maui, Hawaii, USA: ACM 53 58

[B19] Kilicoglu H Shin D Fiszman M Rosemblat G Rindflesch TC Shin D Fiszman M Rosemblat G Rindflesch TC SemMedDB: a PubMed-scale repository of biomedical semantic predications Bioinformatics 2012 28 23 3158 3160 10.1093/bioinformatics/bts591 23044550 PMC3509487

[B20] Knox C Law V Jewison T Liu P Ly S Frolkis A Pon A Banco K Mak C Neveu V Djoumbou Y Eisner R Guo AC Wishart DS Law V Jewison T Liu P Ly S Frolkis A Pon A Banco K Mak C Neveu V Djoumbou Y Eisner R Guo AC Wishart DS DrugBank 3.0: a comprehensive resource for ‘omics’ research on drugs Nucleic Acids Res 2011 39 Database issue D1035 D1041 21059682 10.1093/nar/gkq1126 PMC3013709

[B21] Milo R Shen-Orr S Itzkovitz S Kashtan N Chklovskii D Alon U Network motifs: simple building blocks of complex networks Science 2002 298 5594 824 827 10.1126/science.298.5594.824 12399590

[B22] Alon U Network motifs: theory and experimental approaches. Nature reviews Genetics 2007 8 6 450 461 17510665 10.1038/nrg2102

[B23] Wuchty S Oltvai ZN Barabasi AL Evolutionary conservation of motif constituents in the yeast protein interaction network Nat Genet 2003 35 2 176 179 10.1038/ng1242 12973352

[B24] Yeger-Lotem E Sattath S Kashtan N Itzkovitz S Milo R Pinter RY Alon U Margalit H Sattath S Kashtan N Itzkovitz S Milo R Pinter RY Alon U Margalit H Network motifs in integrated cellular networks of transcription-regulation and protein-protein interaction Proc Natl Acad Sci U S A 2004 101 16 5934 5939 10.1073/pnas.0306752101 15079056 PMC395901

[B25] Masoudi-Nejad A Schreiber F Kashani ZR Building blocks of biological networks: a review on major network motif discovery algorithms IET Syst Biol 2012 6 5 164 174 10.1049/iet-syb.2011.0011 23101871

[B26] Wernicke S Rasche F FANMOD: a tool for fast network motif detection Bioinformatics 2006 22 9 1152 1153 10.1093/bioinformatics/btl038 16455747

[B27] Barabasi AL Oltvai ZN Network biology: understanding the cell’s functional organization. Nature reviews Genetics 2004 5 2 101 113 14735121 10.1038/nrg1272

[B28] Dobrin R Beg QK Barabasi AL Oltvai ZN Beg QK Barabasi AL Oltvai ZN Aggregation of topological motifs in the Escherichia coli transcriptional regulatory network BMC Bioinformatics 2004 5 10 10.1186/1471-2105-5-10 15018656 PMC357809

[B29] Kilicoglu H Beg QK Barabasi AL Oltvai ZN Beg QK Barabasi AL Oltvai ZN Semantic MEDLINE: a Web application to manage the results of PubMed searches 2008 Biomedicine: Proceeings of the Third International Symposium for Semantic Mining in 69 76

[B30] Stelzl U Worm U Lalowski M Haenig C Brembeck FH Goehler H Stroedicke M Zenkner M Schoenherr A Koeppen S Timm J Mintzlaff S Abraham C Bock N Kietzmann S Goedde A Toksoz E Droege A Krobitsch S Korn B Birchmeier W Lehrach H Wanker EE A human protein-protein interaction network: a resource for annotating the proteome Cell 2005 122 6 957 968 10.1016/j.cell.2005.08.029 16169070

[B31] Igor U Krishnamurthy A Karp RM Shamir R Krishnamurthy A Karp RM Shamir R DEGAS: De Novo Discovery of Dysregulated Pathways in Human Diseases PLoS One 2010 5 10 10.1371/journal.pone.0013367 PMC2957424 20976054

[B32] Ideker T Sharan R Protein networks in disease Genome Res 2008 18 4 644 652 10.1101/gr.071852.107 18381899 PMC3863981

[B33] Kuypers DR Skin problems in chronic kidney disease. Nature clinical practice Nephrology 2009 5 3 157 170 19190625 10.1038/ncpneph1040

[B34] Dalhoff K Faerber P Arnholdt H Sack K Strubelt O Faerber P Arnholdt H Sack K Strubelt O [Acute kidney failure during psoriasis therapy with fumaric acid derivatives] Dtsch Med Wochenschr 1990 115 26 1014 1017 10.1055/s-2008-1065114 2361438

[B35] Talamo BR Rudel R Kosik KS Lee VM Neff S Adelman L Kauer JS Pathological changes in olfactory neurons in patients with Alzheimer’s disease Nature 1989 337 6209 736 739 10.1038/337736a0 2465496

[B36] Cardinale D Colombo A Sandri MT Lamantia G Colombo N Civelli M Martinelli G Veglia F Fiorentini C Cipolla CM Colombo A Sandri MT Lamantia G Colombo N Civelli M Martinelli G Veglia F Fiorentini C Cipolla CM Prevention of high-dose chemotherapy-induced cardiotoxicity in high-risk patients by angiotensin-converting enzyme inhibition Circulation 2006 114 23 2474 2481 10.1161/CIRCULATIONAHA.106.635144 17101852

[B37] Zhang Y Xuan J Reyes BG d l Clarke R Ressom HW Xuan J Reyes BG d l Clarke R Ressom HW Network motif-based identification of transcription factor-target gene relationships by integrating multi-source biological data BMC Bioinformatics 2008 9 203 10.1186/1471-2105-9-203 18426580 PMC2386822

[B38] Zhang Y Xuan J de Los Reyes BG Clarke R Ressom HW Network motif-based identification of breast cancer susceptibility genes. in Annual International Conference of the IEEE Engineering in Medicine and Biology Society 2008 5696 5699 10.1109/IEMBS.2008.4650507 19164010

[B39] Goel R Harsha HC Pandey A Prasad TS Harsha HC Pandey A Prasad TS Human protein reference database and human proteinpedia as resources for phosphoproteome analysis Mol Biosyst 2012 8 2 453 463 10.1039/c1mb05340j 22159132 PMC3804167

[B40] Franceschini A Harsha HC Pandey A Prasad TS STRING v9.1: protein-protein interaction networks, with increased coverage and integration Nucleic Acids Res 2013 41 Database issue D808 D815 23203871 10.1093/nar/gks1094 PMC3531103

