# Supplementary material for: Network-based analysis reveals distinct association patterns in a semantic MEDLINE-based drug-disease-gene network
Source: J Biomed Semantics. 2014 Aug 6;5:33. doi: 10.1186/2041-1480-5-33 (PMC4137727; doi:10.1186/2041-1480-5-33)
Supplement: Additional file 4 — NM analysis of drug-gene, disease-drug, and gene-drug networks. [file 2041-1480-5-33-S4.docx]

**Drug-gene network**

| **ID** | **Adj-Matrix** | **Frequency [Original]** | **Mean-Freq [Random]** | **Standard-Dev [Random]** | **Z-Score** | **p-Value** |
| --- | --- | --- | --- | --- | --- | --- |
| 238 | 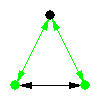 | 0.062426% | 0.039488% | 3.2902e-005 | 6.9717 | <0.001 |

**Gene-disease network**

| **ID** | **Adj-Matrix** | **Frequency [Original]** | **Mean-Freq [Random]** | **Standard-Dev [Random]** | **Z-Score** | **p-Value** |
| --- | --- | --- | --- | --- | --- | --- |
| 238 | 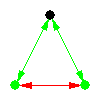 | 0.053754% | 0.02662% | 5.11e-005 | 5.31 | <0.001 |
| 238 | 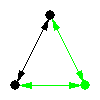 | 0.066609% | 0.025705% | 7.9663e-005 | 5.1347 | 0.001 |
| 78 | 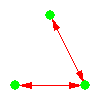 | 0.064271% | 0.040913% | 0.00010186 | 2.2931 | 0.025 |

**Drug-gene network**

No significant results were found.
